# *Blastocystis* Colonization Is Associated with Increased Diversity and Altered Gut Bacterial Communities in Healthy Malian Children

**DOI:** 10.3390/microorganisms7120649

**Published:** 2019-12-04

**Authors:** Aly Kodio, Drissa Coulibaly, Abdoulaye Kassoum Koné, Salimata Konaté, Safiatou Doumbo, Abdoulaye Guindo, Fadi Bittar, Frédérique Gouriet, Didier Raoult, Mahamadou Aly Thera, Stéphane Ranque

**Affiliations:** 1 Aix Marseille Université, Institut de Recherche pour le Développement, Assistance Publique-Hôpitaux de Marseille, Service de Santé des Armées, VITROME: Vecteurs—Infections Tropicales et Méditerranéennes, 19-21 Boulevard Jean Moulin, 13005 Marseille, France; alykodio81@gmail.com; 2 IHU Méditerranée Infection, 19-21 Bd Jean Moulin, 13005 Marseille, France; fadi.BITTAR@univ-amu.fr (F.B.); frederique.gouriet@ap-hm.fr (F.G.); didier.raoult@gmail.com (D.R.); 3 Malaria Research and Training Centre-International Center for Excellence in Research (MRTC-ICER), Department of Epidemiology of Parasitic Diseases, Faculty of Medicine and Dentistry, Université des Sciences, des Techniques et des Technologies de Bamako, Point G, BP 1805 Bamako, Mali; coulibalyd@icermali.org (D.C.); fankone@icermali.org (A.K.K.); sakonate@icermali.org (S.K.); sdoumbo@icermali.org (S.D.); abdouguindo@icermali.org (A.G.); mthera@icermali.org (M.A.T.); 4 Aix Marseille Université, Institut de Recherche pour le Développement, Assistance Publique-Hôpitaux de Marseille, Service de Santé des Armées, MEPHI: Microbes, Evolution, Phylogénie et Infection, 19-21 Boulevard Jean Moulin, 13005 Marseille, France

**Keywords:** *Blastocystis*, healthy children, diversity, bacterial gut microbiota

## Abstract

*Blastocystis* is the most common protozoan colonizing the gut of vertebrates. It modulates the human digestive microbiota in the absence of inflammation and gastrointestinal disease. Although it has been associated with human diseases, including inflammatory bowel disease, its pathogenicity remains controversial. This study aimed to assess the influence of *Blastocystis* on the gut bacterial communities in healthy children. We conducted a cross-sectional study on 147 *Blastocystis*-colonized and 149 *Blastocystis*-noncolonized Malian children, with *Blastocystis* colonization assessed by real-time PCR and gut microbial communities characterized via 16S rRNA gene (Illumina MiSeq) sequencing and bioinformatics analysis. The gut microbiota diversity was higher in *Blastocystis*-colonized compared to *Blastocystis*-noncolonized children. The phyla Firmicutes, Elusimicrobia, Lentisphaerae, and Euryarchaeota were higher in *Blastocystis*-colonized children, whereas Actinobacteria, Proteobacteria, unassigned bacteria, and Deinococcus–Thermus were higher in *Blastocystis*-noncolonized children. Moreover, *Faecalibacterium prausnitzii* (family Ruminococcaceae) and *Roseburia* sp. (family Lachnospiraceae) abundance was higher in *Blastocystis*-colonized children. We conclude that *Blastocystis* colonization is significantly associated with a higher diversity of the gut bacterial communities in healthy children, while it is not associated with the presence of potentially pathogenic bacteria in the human gut.

## 1. Introduction

*Blastocystis* is a genus of unicellular protozoan of the stramenopile group in the eukaryotic domain, described in 1911 by Alexeieff [1]. Many types of *Blastocystis* live in anaerobic conditions within the gut of humans as well as that of various animals, including mammals, birds, reptiles, amphibians, and insects [2]. *Blastocystis* are transmitted by ingestion of cysts; this fecal–oral route is fostered by poor hygiene, contact with animals, and fecal contamination of food and water sources [3,4]. *Blastocystis* is the most common parasite of the human gut; its prevalence ranges from 0.5% in Japan to 60% in Malaysia [3], 24% in Denmark and the Netherlands, and 7% in Italy and the United Kingdom [5]. A relatively higher prevalence has been reported in developing countries with relatively poor health care and hygiene; for instance, *Blastocystis* was found to colonize 100% of the children in a rural area of Senegal [6].

*Blastocystis* is present in both asymptomatic and symptomatic hosts; thus, its implication in human diseases remains controversial. It has been associated with gastrointestinal or dermatological symptoms [7,8,9]. The increase in CD4+ T lymphocyte count was found to be inversely associated with the detection rate of intestinal parasites, including *Blastocystis*, while the increase in viral load was found to be positively associated with it [10,11]. *Blastocystis* has been reported as a cause of diarrhea in renal transplant or bone marrow transplant recipients [12,13]. *Blastocystis* has also been pointed out as a possible causative agent of chronic gastrointestinal diseases, such as irritable bowel disease (IBD) and Crohn’s diseases [14,15]. Today, whether *Blastocystis* is a pathogen or a commensal of the human gut remains an open question.

Interestingly, *Blastocystis* is commonly detected in the gut of asymptomatic humans, and it has been shown that colonization is stable over 6–10 years [16,17]. It was shown that *Blastocystis* was more frequent in healthy subjects compared to subjects with IBD, pointing out a possible protective effect of its colonization [18,19,20]. It is known that the gut microbial communities include protozoa, fungi, bacteria, and viruses, which share the same environment and live in close relationship. Their interactions may result in altered microbiota composition, referred to as dysbiosis. The advent of high throughput sequencing and associated bioinformatics analysis tools has generated new tools for the characterization of microbial communities. Yet, this protozoa-associated dysbiosis remains poorly understood. It was shown that bacterial dysbiosis occurred in subjects with chronic diseases, such as IBD, which are associated with *Blastocystis* [21,22]. A study showed a direct association between *Blastocystis* and gut dysbiosis in the absence of gastrointestinal diseases or inflammation [23]. Like *Blastocystis*, other protozoa have been associated with the modulation of the gut bacterial community. For instance, the gut bacterial community of *Cryptosporidium parvum*-infected mice differed from that of uninfected ones [24], and *Giardia duodenalis* altered human biofilms and microbial communities, leading to an increased abundance of Firmicutes (order Clostridiales) and Bacteroidetes in germ-free mice [25]. It was observed that an increase in both *Prevotella copri* and *Entamoeba histolytica* was associated with diarrhea in two-year-old children [26]. Another study reported that *Entamoeba histolytica* infection was associated with decreased Clostridia, *Bacteroides*, *Lactobacillus*, *Campylobacter*, and *Eubacterium* and increased *Bifidobacterium* abundance compared to healthy controls [27].

This study aimed to assess the influence of *Blastocystis* colonization on gut bacterial communities in healthy Malian children. The gut microbial community was characterized by 16S rDNA gene (Illumina MiSeq) sequencing and bioinformatics analysis on 147 *Blastocystis*-colonized and 149 *Blastocystis*-noncolonized children.

## 2. Patients and Methods

### 2.1. Study Population

The inclusion criteria were as follows: (1) part of a cohort study on malaria incidence conducted at the Bandiagara Malaria Project (BMP) clinical research center in Bandiagara, Mali; (2) aged 6 months to 15 years; (3) no fever and a negative thick blood smear for *Plasmodium* spp. upon inclusion; (4) informed consent to participate in the study. Sociodemographic and clinical data (age, gender, ethnicity, antibiotic use, and residence location) were collected for each participant in October 2017. Concomitantly, stool samples were collected from these children in appropriate, sterile, and identified containers. Within 24 h, solid stools were diluted v/v with 10X phosphate-buffered saline (PBS pH 7.4, RNase-free); all stools were aliquoted into 1 mL tubes and stored at −20 °C before being shipped to Marseille.

### 2.2. DNA Extraction

The total stool DNA was extracted with the EZ1 DNA tissue kit (Qiagen GmbH, Hilden, Germany) according to the procedure described herein [28]. Briefly, 200 mg (200 µL if liquid stools) and 350 µL of G2 lysis buffer were added in a 1.5 mL tube containing 200 mg of 2 mm glass powder. The sample was grinded with the FastPrep-24^TM^5G V. 6005.1 (M.P Biomedicals, LLC Santa Ana CA, USA) at 6 m/s for 40 s and then incubated at 100 °C for 10 min. After centrifugation at 10,000 × *g* for 10 min, 200 µL of the supernatant was recovered, and 10 µL of proteinase K was added followed by overnight incubation at 55 °C. We added 10 µL of a synthetic sequence of 142 bp (5′GCTACTGAGTCGTACCTAATGCATGACCTAGAGCACTCGACTGTTTATCAGTGTCGAGACTCGACGCATGCACGTACGAACCTAGCTGTCAGCAATCGCGAATGCCTACTAAGTAGCGAACTTTAGCGAATCGCGATACGAC-3′) as a first extraction control at the concentration of 200 nmol diluted at 10^−10^ in supernatants after the proteinase K digestion. The automated EZ1 Advanced XL (QIAGEN Instruments, Hombrechtikon, Switzerland) with the DNA card bacteria V 1.066069118 QIAGEN and the EZ1 DNA tissue kit was used to obtain 200 µL total DNA according to the procedure of extraction. Real-time PCR was performed on each DNA extracted for amplification of a synthetic sequence using the primers TissF_5′-CTGAGTCGTACCTAATGCATGACC-3′ and TissR_5′-GTATCGCGATTCGCTAAAGTTC-3′ and the probe TissP_6FAM-5′-TCGAGACTCGACGCATGCACG-Tamra-3′. The second extraction control of bacterial DNA was also carried out on the total DNA as described in [29]. The lysis buffer used for the extraction was used for the PCR negative controls. The extracted DNA was kept at 4 °C and immediately used as template for the PCR detection of gastrointestinal eukaryotic pathogens.

### 2.3. Real-Time PCR Assays

The primers and probes detailed in the paper of Sow et al. [30] were used to assay the twenty gastrointestinal eukaryotic pathogens on the CFX96TM and CFX384TM real-time PCR detection systems (BIO-RAD, Life Science, Marnes-la-coquette, France). The amplification reaction included 10 µL of master mix (Roche diagnostics GmbH, Mannheim, Germany), 0.5 µL of each primer, 0.5 µL of probe, 3 µL of distilled water, 0.5 µL of uracil-DNA glycosylase (UDG), and 5 µL of DNA in a total volume of 20 µL. The amplification program was as follows: 2 min at 50 °C, 5 min at 95 °C, followed by 40 cycles of 5 s at 95 °C and 30 s at 60 °C. A parasite-specific plasmid and a mixture of the DNA-free amplification reaction were used as positive and negative controls, respectively. Analyzed samples with no detectable amplification and Ct values greater than 39 were considered negative.

### 2.4. DNA Extraction and 16S Metabarcoding

DNA was extracted from stool samples after a first mechanical lysis step performed with acid-washed powder (≤106 μm) glass beads (G4649-500G Sigma-Aldrich, St. Quentin Fallavier, France) and 0.5 mm glass bead cell disruption media (Scientific Industries Inc., Bohemia, NY, USA) using a FastPrep BIO 101 instrument (Qbiogene, Strasbourg, France) at maximum speed (6.5 m/s) for 90s. Then, the stools were further lysed by two methods: (1) the classical lysis and protease step followed by purification on the NucleoSpin tissue kit (Macherey Nagel, Hoerdt, France) (protocol 1) and (2) deglycosylation and purification on the EZ1 Advanced XL device (Qiagen, Courtaboeuf, France) (protocol 5) [31]. Samples were first amplified on each of these two extraction products, then pooled and barcoded. The 16S rRNA sequencing was performed on the MiSeq system (Illumina, Inc, San Diego, CA, USA) with paired-end strategy, constructed according to the 16S metagenomic sequencing library preparation (Illumina). For each metagenomic DNA extraction protocol, the 16S “V3–V4” regions was amplified by PCR for 45 cycles using the Kapa HiFi Hotstart ReadyMix 2x (Kapa Biosystems Inc, Wilmington, MA, USA) and the surrounding conserved region V3_V4 primers with overhang adapters (FwOvAd_341F TCGTCGGCAGCGTCAGATGTGTATAAGAGACAGCCTACGGGNGGCWGCAG; RevOvAd_785R GTCTCGTGGGCTCGGAGATGTGTATAAGAGACAGGACTACHVGGGTATCTAATCC). After amplicon purification on AMPure beads (Beckman Coulter Inc., Fullerton, CA, USA), DNA concentration was measured using high-sensitivity Qubit technology (Beckman Coulter Inc, Fullerton, CA, USA) and then diluted to 3.5 ng/µL. At this step, the library of protocol 1 was pooled volume to volume with the library of protocol 5 so that 15 ng was involved in a subsequent limited cycle PCR, where Illumina sequencing adapters and dual-index barcodes were added to the amplicon. After purification on AMPure beads (Beckman Coulter Inc, Fullerton, CA, USA), this library was pooled with 95 other multiplexed samples. The global concentration was quantified by a Qubit assay with the high-sensitivity kit (Life technologies, Carlsbad, CA, USA). Before loading for sequencing on a MiSeq system (Illumina Inc., San Diego, CA, USA), the pool was diluted at 8 pM. Automated cluster generation and paired-end sequencing with dual index reads were performed in a single 39 h run in 2 × 250 bp. The paired reads were filtered according to the read qualities. The raw data were configured in FASTAQ files for R1 and R2 reads.

### 2.5. Bioinformatics Analysis

The 16S metabarcode bioinformatic analysis was performed with the MetaGX tool according to the bioinformatic company XEGEN’s protocol [32]. Each sample read was analyzed by the VSEARCH tool to check raw data quality. The data cleaning was mainly done with the MiSeq sequencer software and then with the Cutadapt tool to eliminate sequencing primers and reads that were poor in quality and/or too short. The paired reads were kept and merged by the PANDAseq tool. The QIMME tool was used for merging and labeling the samples and then for clustering and operational taxonomic unit (OTU) creation using 97% of the clustering threshold. The QIMME tool allowed the OTUs to be filtered and a representative sequence for each OTU to be chosen.

The taxonomic assignment for each OTU was performed via BLAST querying the SILVA and the IHU MI culturomics in-house databases. Blast hits were sorted on the basis of coverage and percentage of identity depending on the (1) presence of one or more blast hits associated with a reference sequence (100% coverage; identity >97% corresponds to OTU’s assignment to the species associated with the best blast hit); (2) presence of less relevant blast hits (identity between 95% and 97%: assignment to genus level; between 90% and 95%: assignment to the family; below 90%: assignment to the kingdom) with creation of a putative species in each case; (3) no blast hits (creation of putative new bacterial species).

### 2.6. Statistical Analysis

Multivariate linear regression analysis was used to test the association between *Blastocystis* and sociodemographic and clinical covariates. The number of observed OTUs; the Shannon, Simpson, and Chao-1 diversity indices; the abundance of OTUs; and the Bray–Curtis dissimilarity index were computed with the PAST3 software package (PAleontological Statistics Sofwware Version 3.20 [https://palaeo-electronica.org/2001_1/past/issue1_01.htm]). The median, interquartile range, mean, and standard deviation of these covariates were tabulated. Alpha diversity in *Blastocystis*-colonized and *Blastocystis*-noncolonized by were compared via the Mann–Whitney–Wilcoxon test with the GraphPad Prism software version 7.00 for Windows. Principal coordinate analysis (PCoA) in PAST3 was used to display the association of bacterial communities with *Blastocystis*-colonized or *Blastocystis*-noncolonized children. The bacterial community structure of the two groups was compared using the nonparametric statistical analysis of significant difference between two (permutational multivariate analysis of variance (PERMANOVA)) tests. A linear discriminant effect size (LEfSe) analysis was used to estimate taxon effect values between the two groups (http://huttenhower.sph.harvard.edu/lefse/). All statistical tests were two-sided, and *p* < 0.05 was considered statistically significant.

### 2.7. Ethical Considerations

The study was conducted in accordance with the Declaration of Helsinki, and the protocol was approved by the Ethics Committee of the Faculty of Medicine of Mali (No 2017/133/CE/FMPOS). Written informed consent for participation in the study was obtained from each child and at least one of his/her parents or responsible person, and written assent was obtained from older children.

## 3. Results

### 3.1. Characteristics of Study Subjects

In this study, we included 300 healthy participants from a prospective malaria cohort study with a mean age of 8 years, consisting of 154 (51.3%) females and 146 (48.7%) males (*p* = 0.97). We collected one stool sample from each child. Four samples were excluded, giving a total of 296 (98.7%) samples. Real-time PCR detected 147/296 (49.7%) *Blastocystis*-positive stool samples. The mean age of *Blastocystis*-positive and *Blastocystis*-negative subjects was 7.5 years and 8.3 years, respectively (*p* = 0.32). The male/female sex ratio was 0.97 and 0.89 in *Blastocystis*-positive and *Blastocystis*-negative subjects, respectively (*p* = 0.93).

Multivariate analysis highlighted a statistically significant association of *Blastocystis* colonization with age (*p* = 0.001) and weight (*p* = 0.03). No other sociodemographic, clinical, or biological characteristic was statistically significantly associated with *Blastocystis* colonization (Table S1).

### 3.2. Diversity and Composition of the Microbiota in Stool Samples

High throughput Illumina MiSeq sequencing of the 16S rDNA gene generated a total of 63,614,614 paired reads, of which 61,911,004 high-quality reads were selected for the bioinformatic analysis. The median and mean of the reads were comparable between *Blastocystis*-colonized and *Blastocystis*-noncolonized subjects (*p* = 0.5969) (Supplementary file 1: Table S2). The reads were assigned to 334,703 OTUs and 18 phyla. The predominant phyla were Firmicutes (average 43.23%) and Bacteroidetes (average 10.64%), followed by Actinobacteria (average 9.39%) and Proteobacteria (average 6.86%) (Supplementary file 2: Figure S1). The most abundant classes were Clostridia (34.57%), Bacteroidia (9.37%), Bacilli (8.36%), Actinobacteria (4.99%), and Coriobacteriia (4.66%) (Supplementary file 2: Figure S2). Regarding orders, Clostridiales (32.98%), Bacteroidales (8.94%), Lactobacilles (6.61%), Coriobacteriales (4.4%), and Bifidobacteriales (4.02%) were the most abundant (Supplementary file 2: Figure S3). The most common families were Clostridiaceae (11.21%), Ruminococcaceae (7.29%), Lachnospiraceae (6.51%), Peptostreptococcoceae (5.88%), Prevotellaceae (5.03%), Streptococcaceae (3.79%), Bifidobacteriaceae (3.49%), Bacteroidaceae (3.29%), Eubacteriaceae (3.16%), and Coriobacteriaceae (3.08%) (Supplementary file 2: Figure S4).

**Figure 1:**
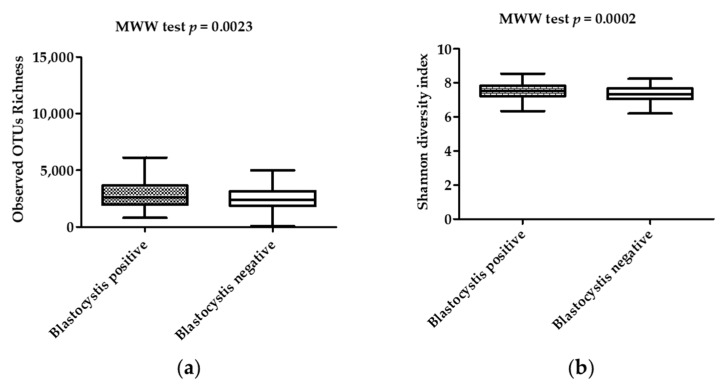
Box plots of (**a**) observed OTU richness and (**b**) Shannon diversity index in *Blastocystis*-colonized and *Blastocystis*-noncolonized children as compared via the Mann–Whitney–Wilcoxon (MWW) test. The interquartile ranges (IQRs, boxes), the median (dark line inside the boxes), and the lowest and highest values within 1.5 times IQR from the first and third quartiles (whiskers above and below the boxes) are plotted for each group.

**Figure 2:**
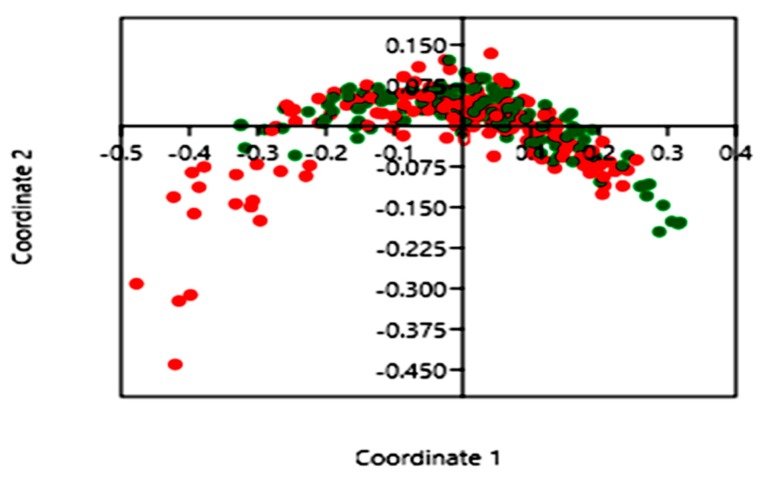
Principal coordinate analysis (PCoA) of the microbial communities in *Blastocystis*-colonized and *Blastocystis*-noncolonized children samples. The *Blastocystis*-colonized children are plotted as green dots and the *Blastocystis*-noncolonized children as red dots.

There was a statistically significantly higher OTU richness and bacterial diversity in *Blastocystis*-colonized compared to *Blastocystis*-noncolonized children. Indeed, both Shannon and Simpson diversity indices showed that the diversity of the gut bacterial communities in *Blastocystis*-noncolonized children was lower than in *Blastocystis*-colonized subjects (*p* < 0.01) (Figure 1; Table 1). In contrast, both the Chao-1 and OTU richness indices showed that the richness of the gut bacterial communities were higher in *Blastocystis*-colonized children compared to *Blastocystis*-noncolonized children (*p* < 0.01) (Figure 1; Table 1). The Bray–Curtis dissimilarity index, which assessed the differences in bacterial community structure between *Blastocystis*-colonized and *Blastocystis*-noncolonized groups of children, was used in PCoA and PERMANOVA. The PCoA showed a clustering of the samples depending on the *Blastocystis* colonization status of the children; coordinate 1 (42.5%) and coordinate 2 (8.7%) scores explained 50% of the variance of the data (Figure 2). Furthermore, PERMANOVA showed a statistically significant difference in the bacterial community structure between the two groups (*p* = 0.0001).

### 3.3. Impact of Blastocystis on Gut Bacterial Communities

The heterogeneity of bacterial communities between *Blastocystis*-colonized and *Blastocystis*-noncolonized children was evaluated via LEfSe [33]. The results showed that the phyla Firmicutes, Elusimicrobia, Lentisphaerae, Euryarchaeota, and IHU_PP_Bacteria were significantly more abundant in the *Blastocystis*-colonized group than Actinobacteria, Proteobacteria, unassigned bacteria, and Deinococcus–Thermus, which were overrepresented in the *Blastocystis*-noncolonized children (Supplementary file 2: Figure S5A).

Regarding bacteria class distribution, a higher trend of Clostridia, IHU_PC_PC_Bacteria, Elusimicrobia, Lentisphaeria, Metanobacteria, and Deltaproteobacteria were observed in the *Blastocystis*-colonized children, whereas Planctomycetacia, Rubrobacteria, Deinococci, Gammaproteobacteria, Actinobacteria, unassigned bacteria, and Bacilli were predominant in the *Blastocystis*-noncolonized children (Supplementary file 2: Figure S5B).

At the order level, the abundance of Clostridiales, IHU_PO_Bacteria, Victivallales, Methanobacteriales, Elusimicrobiales, Aeromonadales, Acidaminococcales, and Desulfovibrionales were more abundant in *Blastocystis*-colonized children, whereas Planctomycetales, Rhodobacterales, Sphingomonadales, Rubrobacterales, Veillonellales, Pasteurellales, Micrococcales, Pseudonocardiales, Enterobacteriales, Myxococcales, Bifidobacteriales, unassigned bacteria, and Lactobacillales were more abundant in *Blastocystis*-noncolonized children (Supplementary file 2: Figure S5C).

At the family level, abundance of Clostridiaceae, Ruminococcaceae, and Lachnospiraceae were higher in *Blastocystis*-colonized children, whereas Streptococcaceae, Bifidobacteriaceae, Enterobacteriaceae, and Leuconostocaceae were higher in *Blastocystis*-noncolonized children (Supplementary file 2: Figure S5D).

At the genus level, *Ruminococcus* and *Clostridium* were among the most abundant genera in the *Blastocystis*-colonized children, whereas *Streptococcus*, *Bifidobacterium*, and *Shigella* were more abundant in *Blastocystis*-noncolonized children (Supplementary file 2: Figure S5E).

At the species level, *Clostridium saudii*, *Methanobrevibacter smithii*, and a few other species were the most abundant in the *Blastocystis*-colonized children, whereas more *Streptococcus* sp., *Bifidobacterium* sp., *Shigella* sp., and a few other species were the most abundant in *Blastocystis*-noncolonized children (*p* < 0.05). Some species were notably abundant in the group of *Blastocystis*-noncolonized children compared to *Blastocystis*-colonized children (*p* < 0.05). (Supplementary file2: Figure S5F).

## 4. Discussion

This study’s main finding was that both diversity and richness of gut bacterial communities was higher in healthy *Blastocystis*-colonized children compared to *Blastocystis*-noncolonized ones. A limitation of our study was that the influence of the children’s lifestyle and diet on the bacterial communities was not assessed. The major strength of our study, compared with previous ones [34], was the robustness of our findings, which was supported by the relatively large sample size of 296 healthy Malian children.

It has been reported that the diversity of the eukaryotic gut communities in healthy humans is of relatively low abundance, stable over time, and dominated by *Blastocystis* subtypes [17]. The influence of *Blastocystis* on the gut bacterial communities has been assessed in several studies [34,35,36,37,38,39,40]. In line with our findings, many studies [34,39,41] have found that *Blastocystis* colonization is associated with a relatively increased diversity of gut bacterial communities. In particular, the diversity was relatively higher among Swedish travelers colonized with *Blastocystis*, which was also associated with both a “healthy” gut microbiota profile and a vegetable-rich diet [37]. Furthermore, a high bacterial community diversity has been observed in *Blastocystis*-colonized patients [34]. A study evaluating the influence of *Giardia duodenalis*, *Entamoeba* spp., and *Blastocystis hominis* infections on the structure of bacterial communities in symptomatic and asymptomatic subjects (adults/infants) in Côte d’Ivoire found that the *Faecalibacterium prausnitzii*/*Escherichia coli* ratio increased in the subjects carrying *Entamoeba* spp. and *Blastocystis hominis* compared to those with no enteric protozoan [39]. In contrast, the diversity of bacterial community decreased in *Blastocystis*-colonized patients with hepatic encephalopathy [35]. However, a decreased bacterial diversity has been observed in metabolic or infectious diseases, such as IBD or infection with enteric pathogens, which are associated with inflammation of the lower gastrointestinal tract [21,22,42].

Our study showed that the phyla Firmicutes and Bacteroidetes were most abundant followed by Actinobacteria and Proteobacteria in all children. This trend was confirmed in a study investigating the association of *Blastocystis* carried by patients with irritable bowel syndrome (IBS) with bacterial communities [38]. The differences in the bacterial community structure of *Blastocystis*-colonized and *Blastocystis*-noncolonized children explained 50% of the variance in a coordinated main component analysis. Furthermore, there was a statistically significant difference (*p* = 0.0001) using a nonparametric similarity test (PERMANOVA) with the Bray–Curtis similarity measure, supporting the disparity of the bacterial community structures in *Blastocystis*-colonized and *Blastocystis*-noncolonized children. Similarly, Audebert et al. found that *Blastocystis* colonization explained 30% of the variance in bacterial community structure, which was statistically significant (*p* = 0.0001) via PERMANOVA [34].

High-throughput Illumina MiSeq sequencing technology was used to characterize the gut bacterial community structure of the *Blastocystis*-colonized and *Blastocystis*-noncolonized children. The LEfSe, designed to discriminate the taxa associated with each group, highlighted a significant increase in the phyla Firmicutes, Elusimicrobia, Lentisphaerae, and Euryarchaeota in *Blastocystis*-colonized children, while Actinobacteria, Proteobacteria, unassigned bacteria, and Deinococcus–Thermus were higher in *Blastocystis*-noncolonized children. Therefore, *Blastocystis* colonization was associated with eubiosis, a condition characterized by a higher proportion of “beneficial bacteria” (Firmicutes and Bacteroides) than “probable pathogenic bacteria” (Proteobacteria). The authors hypothesized that *Blastocystis* colonization could be a surrogate marker of a “healthy gut microbiota”. In line with this proposition, it has been reported that *Blastocystis* colonization is more frequent in healthy subjects compared to patients with IBD [18,19].

Furthermore, we found higher bacterial species diversity in *Blastocystis*-colonized compared to *Blastocystis*-noncolonized children. LEfSe highlighted that Clostridiaceae, Ruminococcaceae, and Lachnospiraceae were more abundant in *Blastocystis*-colonized children, whereas Streptococcaceae, Bifidobacteriaceae, Enterobacteriaceae, and Leuconostocaceae were more abundant in *Blastocystis*-noncolonized children. More precisely, *Faecalibacterium prausnitzii* (family Ruminococcaceae) and *Roseburia* sp. (family Lachnospiraceae) were relatively more abundant in children colonized by *Blastocystis*. This is of particular interest because other authors have reported that *Faecalibacterium prausnitzii* and *Roseburia* sp. abundance is decreased in the gut bacterial community of Crohn’s disease patients [43]. Both *Faecalibacterium prausnitzii* and *Roseburia* sp. play an important protective role in gut physiology. They digest dietary fiber into short-chain fatty acids, especially butyrate, which provides an energy source for intestinal cells [44]. They also possess anti-inflammatory properties [45,46,47]. Similar to our findings, another study [34] showed that *Faecalibacterium prausnitzii* and *Roseburia* sp. were relatively more abundant in patients colonized by *Blastocystis*; this suggests that *Blastocystis* colonization could be used as a surrogate marker of a healthy gut microbiota.

## 5. Conclusions

*Blastocystis* colonization was significantly associated with a higher diversity and richness of the gut bacterial communities in healthy children. Also, *Blastocystis* colonization was associated with a higher proportion of “beneficial bacteria” (Firmicutes and Bacteroides) than “probable pathogenic bacteria” (Proteobacteria) in the human gut. Indeed, because of its hypothetical capacity to promote, or at least be associated with, an increased diversity of gut bacterial communities, documenting the presence of *Blastocystis* within a healthy microbiota donor would be critical in the management of patients whose diseases require fecal transplantation. Further studies aimed at elucidating the mechanisms by which *Blastocystis* influences the gut bacterial communities are warranted.

## Figures and Tables

**Table 1:** Bacterial community richness and diversity indices in *Blastocystis*-colonized and *Blastocystis*-noncolonized children. The diversity and richness indices between the two groups were compared using the Mann–Whitney–Wilcoxon test. OTUs: operational taxonomic units.

*Blastocystis* Status of the Children			Colonized	Noncolonized	*p* Value
Bacterial community richness	Observed OTUs	Mean (standard deviation)	3008 (1496)	2476 (1079)	0.0023
		Median (interquartile range)	2622 (1951–3630)	2378 (1839–3138)	
	Chao-1 index	Mean (standard deviation)	9966 (6104)	8021 (4321)	0.007
		Median (interquartile range)	8391 (5573–12300)	6824 (4833–10375)	
Bacterial diversity	Shannon index	Mean (standard deviation)	7.536 (0.4698)	7.299 (0.5710)	0.0002
		Median (interquartile range)	7.511 (7.238–7.072)	7.324 (7.833–7.683)	
	Simpson index	Mean (standard deviation)	0.9991 (0.0005267)	0.9987 (0.001515)	<10^−4^
		Median (interquartile range)	0.9992 (0.9988–0.9995)	0.9990 (0.9985–0.9994)	

## Supplementary Materials

The following are available online at https://www.mdpi.com/2076-2607/7/12/649/s1. 16S metabarcoding data and metadata are available online at www.mediterranee-infection.com/acces-ressources/donnees-pour-articles/blastocystis-16s-metabarcoding/.

## References

[1] Lynch K.M. (1916). Dauercystformation of *Trichomonas intestinalis*. J. Parasitol..

[2] Silberman J.D., Sogin M.L., Leipe D.D., Clark C.G. (1996). Human parasite finds taxonomic home. Nature.

[3] Tan K.S.W. (2008). New Insights on Classification, Identification, and Clinical Relevance of *Blastocystis* spp.. Clin. Microbiol. Rev..

[4] Clark C.G., van der Giezen M., Alfellani M.A., Stensvold C.R., Rollinson D. (2013). Chapter One—Recent Developments in *Blastocystis* Research. Advances in Parasitology.

[5] El Safadi D., Cian A., Nourrisson C., Pereira B., Morelle C., Bastien P., Bellanger A.-P., Botterel F., Candolfi E., Desoubeaux G. (2016). Prevalence, risk factors for infection and subtype distribution of the intestinal parasite *Blastocystis* sp. from a large-scale multi-center study in France. BMC Infect. Dis..

[6] El Safadi D., Gaayeb L., Meloni D., Cian A., Poirier P., Wawrzyniak I., Delbac F., Dabboussi F., Delhaes L., Seck M. (2014). Children of Senegal River Basin show the highest prevalence of *Blastocystis* sp. ever observed worldwide. BMC Infect. Dis..

[7] Bálint A., Dóczi I., Bereczki L., Gyulai R., Szűcs M., Farkas K., Urbán E., Nagy F., Szepes Z., Wittmann T. (2014). Do not forget the stool examination!—Cutaneous and gastrointestinal manifestations of *Blastocystis* sp. infection. Parasitol. Res..

[8] Chandramathi S., Suresh K., Sivanandam S., Kuppusamy U.R. (2014). Stress Exacerbates Infectivity and Pathogenicity of *Blastocystis* hominis: In Vitro and In Vivo Evidences. PLoS ONE.

[9] Chandramathi S., Suresh K., Shuba S., Mahmood A., Kuppusamy U.R. (2010). High levels of oxidative stress in rats infected with *Blastocystis hominis*. Parasitology.

[10] Akgül Ö., Kart Yaşar K., Sapmaz B., Kırkoyun Uysal H., Yıldırmak T., Şimşek F., Karasakal Ö.F., Çalışkan R., Öner Y.A. (2018). [Detection of intestinal parasites with conventional and molecular methods in follow-up HIV/AIDS cases]. Mikrobiyol. Bul..

[11] Cirioni O., Giacometti A., Drenaggi D., Ancarani F., Scalise G. (1999). Prevalence and clinical relevance of *Blastocystis hominis* in diverse patient cohorts. Eur. J. Epidemiol..

[12] Ghosh K., Ayyaril M., Nirmala V. (1998). Acute GVHD involving the gastrointestinal tract and infestation with *Blastocystis hominis* in a patient with chronic myeloid leukaemia following allogeneic bone marrow transplantation. Bone Marrow Transplant..

[13] Rao K., Sekar U., Iraivan K.T., Abraham G., Soundararajan P. (2003). *Blastocystis hominis*—An emerging cause of diarrhoea in renal transplant recipients. J. Assoc. Physicians India.

[14] Shariati A., Fallah F., Pormohammad A., Taghipour A., Safari H., Chirani A. salami, Sabour S., Alizadeh-Sani M., Azimi T. (2019). The possible role of bacteria, viruses, and parasites in initiation and exacerbation of irritable bowel syndrome. J. Cell. Physiol..

[15] Khademvatan S., Masjedizadeh R., Rahim F., Mahbodfar H., Salehi R., Yousefi-Razin E., Foroutan M. (2017). Blastocystis and irritable bowel syndrome: Frequency and subtypes from Iranian patients. Parasitol. Int..

[16] Scanlan P.D., Stensvold C.R., Rajilić-Stojanović M., Heilig H.G.H.J., De Vos W.M., O’Toole P.W., Cotter P.D. (2014). The microbial eukaryote *Blastocystis* is a prevalent and diverse member of the healthy human gut microbiota. FEMS Microbiol. Ecol..

[17] Scanlan P.D., Marchesi J.R. (2008). Micro-eukaryotic diversity of the human distal gut microbiota: Qualitative assessment using culture-dependent and-independent analysis of faeces. ISME J..

[18] Petersen A.M., Stensvold C.R., Mirsepasi H., Engberg J., Friis-Møller A., Porsbo L.J., Hammerum A.M., Nordgaard-Lassen I., Nielsen H.V., Krogfelt K.A. (2013). Active ulcerative colitis associated with low prevalence of *Blastocystis* and Dientamoeba fragilis infection. Scand. J. Gastroenterol..

[19] Krogsgaard L.R., Engsbro A.L., Stensvold C.R., Nielsen H.V., Bytzer P. (2015). The Prevalence of Intestinal Parasites Is Not Greater Among Individuals with Irritable Bowel Syndrome: A Population-based Case-control Study. Clin. Gastroenterol. Hepatol..

[20] Rossen N.G., Bart A., Verhaar N., van Nood E., Kootte R., de Groot P.F., D’Haens G.R., Ponsioen C.Y., van Gool T. (2015). Low prevalence of *Blastocystis* sp. in active ulcerative colitis patients. Eur. J. Clin. Microbiol. Infect. Dis..

[21] Lyra A., Lahtinen S. (2012). Dysbiosis of the Intestinal Microbiota in IBS. Current Concepts in Colonic Disorders.

[22] Manichanh C., Borruel N., Casellas F., Guarner F. (2012). The gut microbiota in IBD. Nat. Rev. Gastroenterol. Hepatol..

[23] Nieves-Ramírez M.E., Partida-Rodríguez O., Laforest-Lapointe I., Reynolds L.A., Brown E.M., Valdez-Salazar A., Morán-Silva P., Rojas-Velázquez L., Morien E., Parfrey L.W. (2018). Asymptomatic Intestinal Colonization with Protist *Blastocystis* Is Strongly Associated with Distinct Microbiome Ecological Patterns. mSystems.

[24] Ras R., Huynh K., Desoky E., Badawy A., Widmer G. (2015). Perturbation of the intestinal microbiota of mice infected with Cryptosporidium parvum. Int. J. Parasitol..

[25] Beatty J.K., Akierman S.V., Motta J.-P., Muise S., Workentine M.L., Harrison J.J., Bhargava A., Beck P.L., Rioux K.P., McKnight G.W. (2017). Giardia duodenalis induces pathogenic dysbiosis of human intestinal microbiota biofilms. Int. J. Parasitol..

[26] Gilchrist C.A., Petri S.E., Schneider B.N., Reichman D.J., Jiang N., Begum S., Watanabe K., Jansen C.S., Elliott K.P., Burgess S.L. (2016). Role of the Gut Microbiota of Children in Diarrhea Due to the Protozoan Parasite *Entamoeba histolytica*. J. Infect. Dis..

[27] Burgess S.L., Petri W.A. (2016). The Intestinal Bacterial Microbiome and E. histolytica Infection. Curr. Trop. Med. Rep..

[28] Menu E., Mary C., Toga I., Raoult D., Ranque S., Bittar F. (2018). Evaluation of two DNA extraction methods for the PCR-based detection of eukaryotic enteric pathogens in fecal samples. BMC Res. Notes.

[29] Dridi B., Henry M., Khéchine A.E., Raoult D., Drancourt M. (2009). High Prevalence of Methanobrevibacter smithii and Methanosphaera stadtmanae Detected in the Human Gut Using an Improved DNA Detection Protocol. PLoS ONE.

[30] Sow D., Parola P., Sylla K., Ndiaye M., Delaunay P., Halfon P., Camiade S., Dieng T., Tine R.C.K., Faye B. (2017). Performance of Real-Time Polymerase Chain Reaction Assays for the Detection of 20 Gastrointestinal Parasites in Clinical Samples from Senegal. Am. J. Trop. Med. Hyg..

[31] Angelakis E., Bachar D., Henrissat B., Armougom F., Audoly G., Lagier J.-C., Robert C., Raoult D. (2016). Glycans affect DNA extraction and induce substantial differences in gut metagenomic studies. Sci. Rep..

[32] XEGEN—The Specialist in High Performance and High Throughput NGS Data Analysis and Functional Annotation. http://xegen.eu/.

[33] Segata N., Izard J., Waldron L., Gevers D., Miropolsky L., Garrett W.S., Huttenhower C. (2011). Metagenomic biomarker discovery and explanation. Genome Biol..

[34] Audebert C., Even G., Cian A., Loywick A., Merlin S., Viscogliosi E., Chabé M., The Blastocystis Investigation Group (2016). Colonization with the enteric protozoa *Blastocystis* is associated with increased diversity of human gut bacterial microbiota. Sci. Rep..

[35] Yildiz S., Doğan İ., Doğruman-Al F., Nalbantoğlu U., Üstek D., Sarzhanov F., Yildirim S. (2016). Association of Enteric Protist *Blastocystis* spp. and Gut Microbiota with Hepatic Encephalopathy. J. Gastrointest. Liver Dis. JGLD.

[36] O’Brien Andersen L., Karim A.B., Roager H.M., Vigsnæs L.K., Krogfelt K.A., Licht T.R., Stensvold C.R. (2016). Associations between common intestinal parasites and bacteria in humans as revealed by qPCR. Eur. J. Clin. Microbiol. Infect. Dis. Off. Publ. Eur. Soc. Clin. Microbiol..

[37] Forsell J., Bengtsson-Palme J., Angelin M., Johansson A., Evengård B., Granlund M. (2017). The relation between *Blastocystis* and the intestinal microbiota in *Swedish travellers*. BMC Microbiol..

[38] Nagel R., Traub R.J., Allcock R.J.N., Kwan M.M.S., Bielefeldt-Ohmann H. (2016). Comparison of faecal microbiota in *Blastocystis*-positive and *Blastocystis*-negative irritable bowel syndrome patients. Microbiome.

[39] Iebba V., Santangelo F., Totino V., Pantanella F., Monsia A., Di Cristanziano V., Di Cave D., Schippa S., Berrilli F., D’Alfonso R. (2016). Gut microbiota related to *Giardia duodenalis*, *Entamoeba* spp. and *Blastocystis hominis* infections in humans from Côte d’Ivoire. J. Infect. Dev. Ctries..

[40] Stensvold C.R., van der Giezen M. (2018). Associations between Gut Microbiota and Common Luminal Intestinal Parasites. Trends Parasitol..

[41] Andersen L.O., Bonde I., Nielsen H.B., Stensvold C.R. (2015). A retrospective metagenomics approach to studying *Blastocystis*. FEMS Microbiol. Ecol..

[42] Pham T.A.N., Lawley T.D. (2014). Emerging insights on intestinal dysbiosis during bacterial infections. Curr. Opin. Microbiol..

[43] Sokol H., Seksik P., Furet J.P., Firmesse O., Nion-Larmurier I., Beaugerie L., Cosnes J., Corthier G., Marteau P., Doré J. (2009). Low counts of Faecalibacterium prausnitzii in colitis microbiota. Inflamm. Bowel Dis..

[44] Tedelind S., Westberg F., Kjerrulf M., Vidal A. (2007). Anti-inflammatory properties of the short-chain fatty acids acetate and propionate: A study with relevance to inflammatory bowel disease. World J. Gastroenterol. WJG.

[45] Brestoff J.R., Artis D. (2013). Commensal bacteria at the interface of host metabolism and the immune system. Nat. Immunol..

[46] Hamer H.M., Jonkers D., Venema K., Vanhoutvin S., Troost F.J., Brummer R.-J. (2008). Review article: The role of butyrate on colonic function. Aliment. Pharmacol. Ther..

[47] Tan J., McKenzie C., Potamitis M., Thorburn A.N., Mackay C.R., Macia L. (2014). The role of short-chain fatty acids in health and disease. Adv. Immunol..

