# Supplementary material for: Blastocystis Colonization Is Associated with Increased Diversity and Altered Gut Bacterial Communities in Healthy Malian Children
Source: Microorganisms. 2019 Dec 4;7(12):649. doi: 10.3390/microorganisms7120649 (PMC6956266; doi:10.3390/microorganisms7120649)
Supplement: Supplementary file 1 [file microorganisms-07-00649-s001.zip › Supplementary file 2.docx]

**Figure S1** : Relative abundance of bacterial at phylum level

**Figure S2**: Relative abundance of bacterial at class level

**Figure S3**: Relative abundance of bacterial at order level

**Figure S4**: Relative abundance of bacterial at family level

1. LEfSe for bacterial Phyla


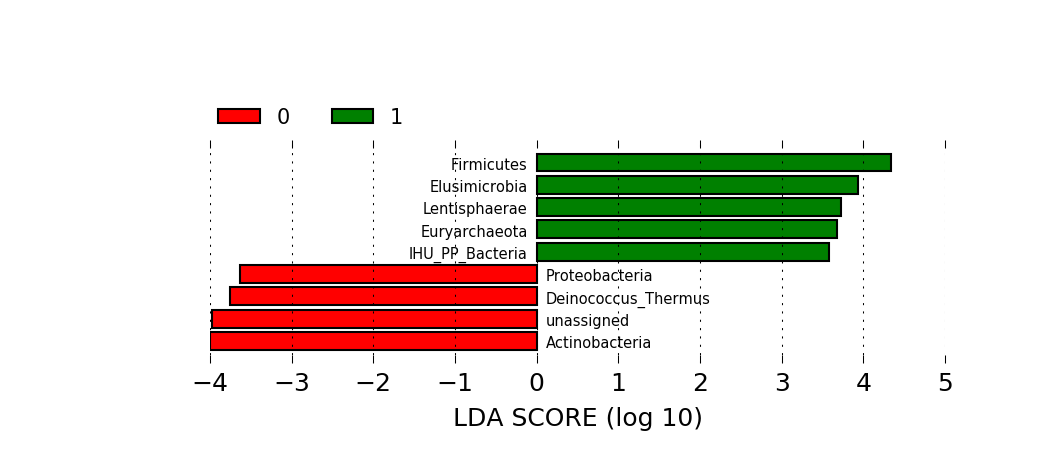


**B)** LEfSe for bacterial Class
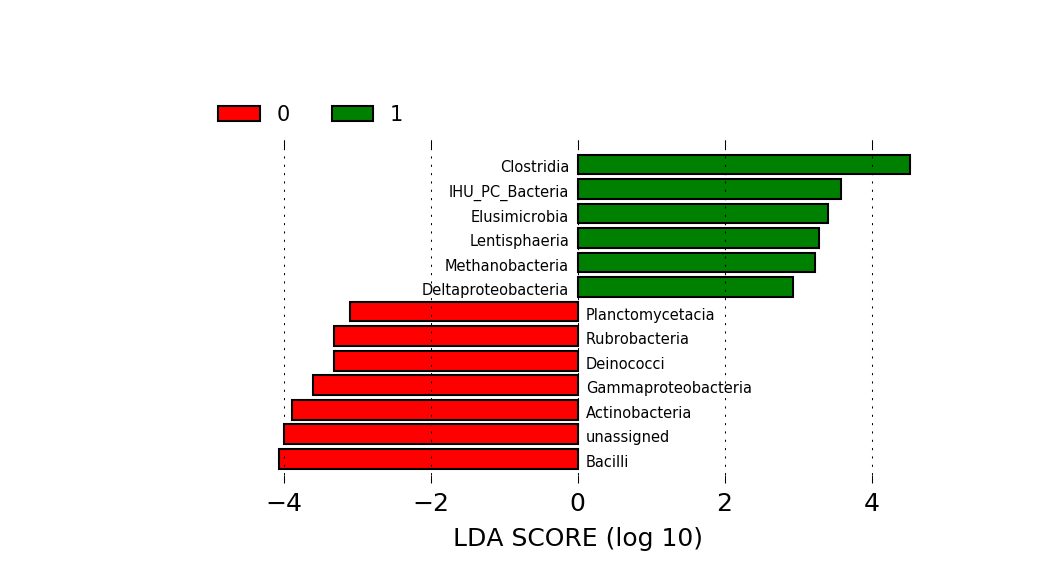


**C)** LEfSe for bacterial Orders
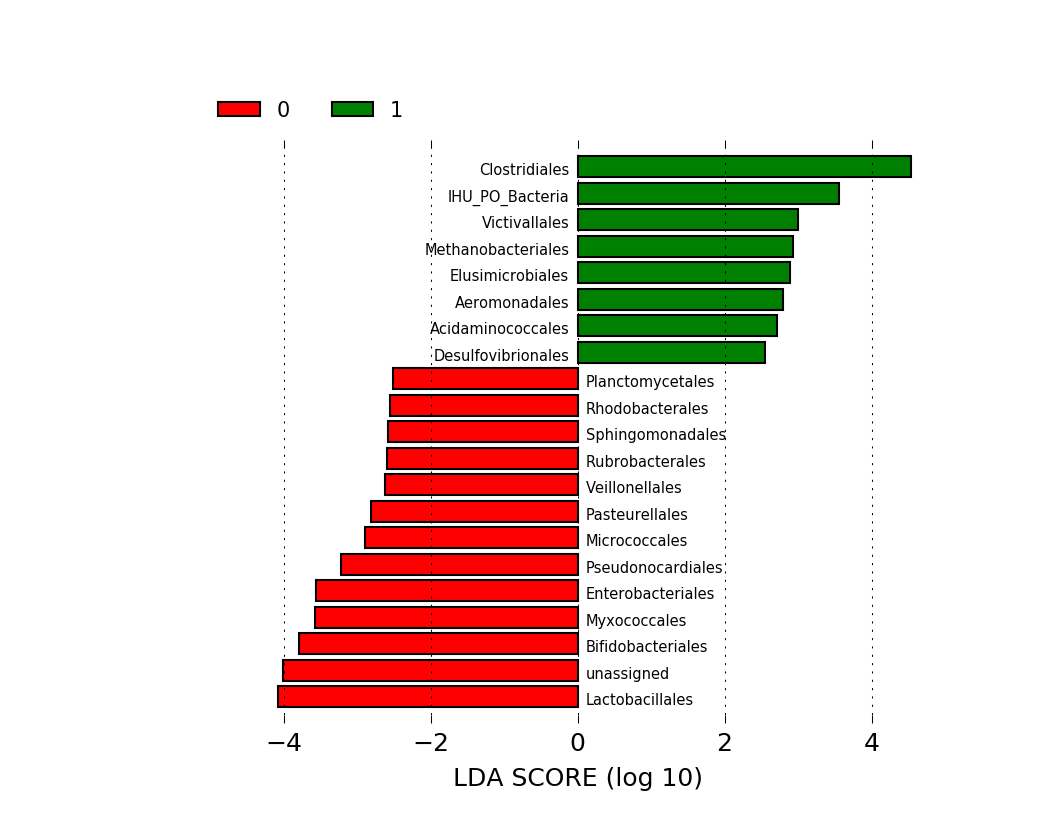


**D)** LEfSe for bacterial Families
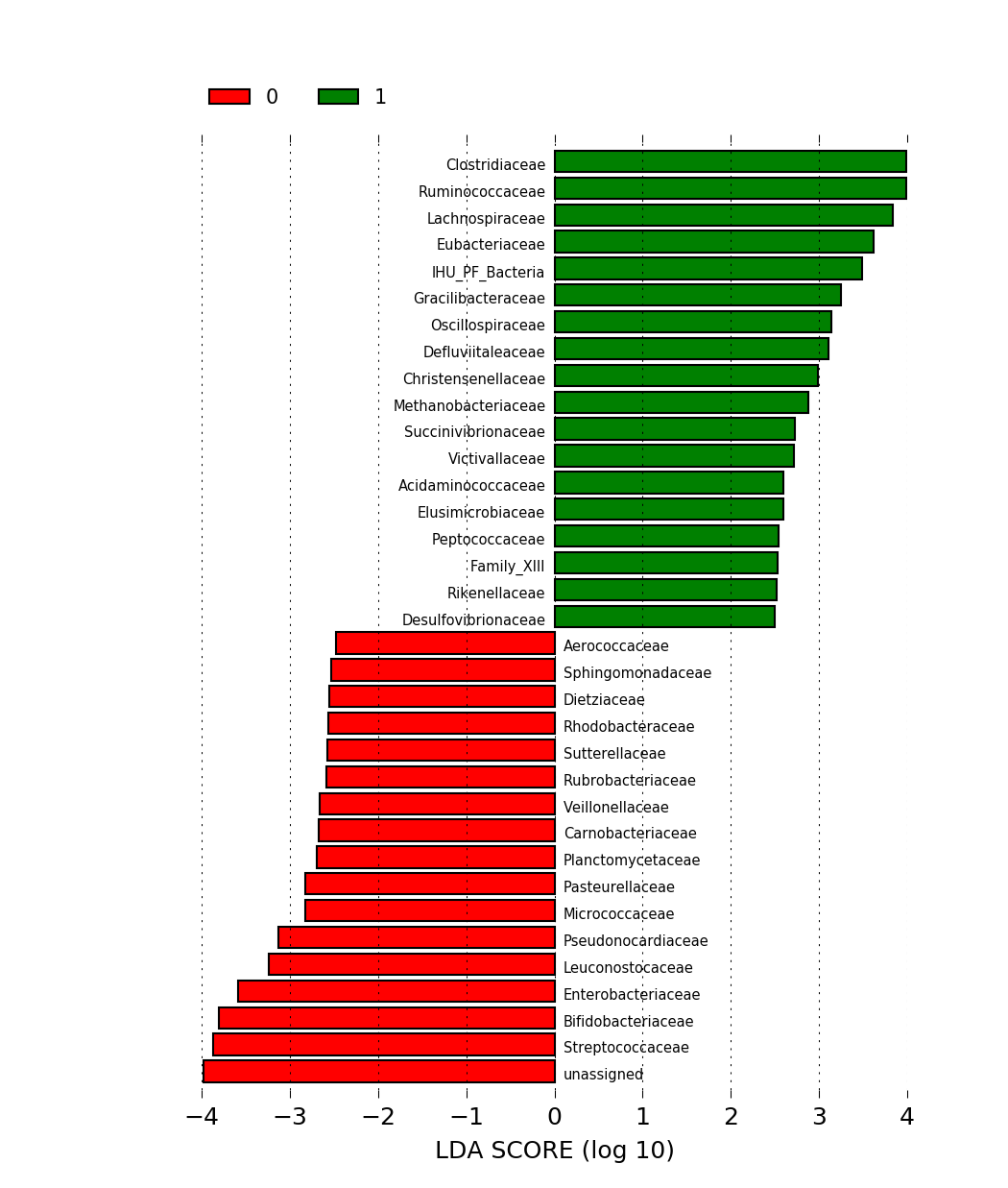


**E)** LEfSe for bacterial Genera


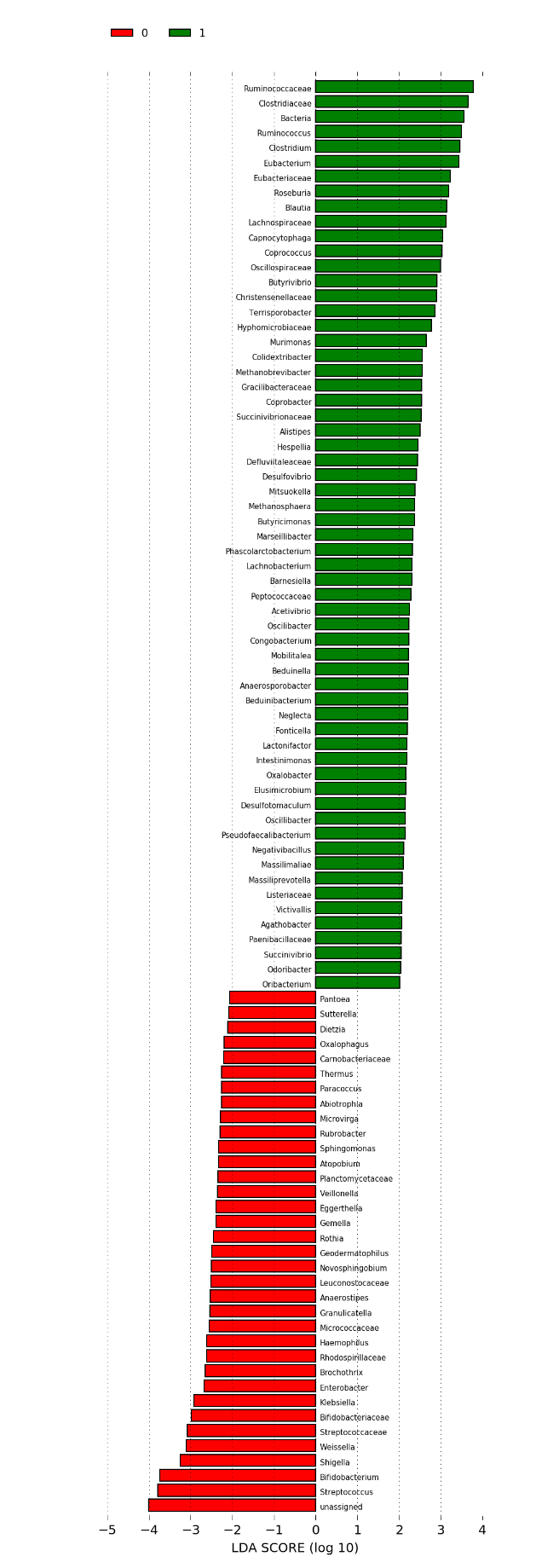


**F)** LEfSe for bacterial Species


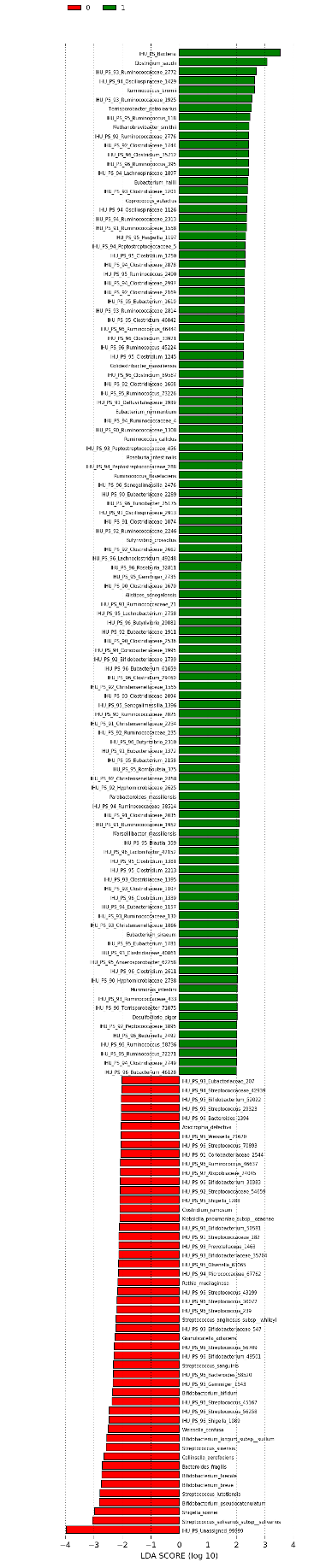


**Figure S5:** Linear Discriminant Analysis LDA Effect Size (LEfSe) performed on microbiota components of *Blastocystis*-colonized (red color) and non-colonized (green color) children. A) LEfSe for the Phyla, B) LEfSe for the Class, C) LEfSe for the Orders, D) LEfSe for the Families, E) LEfSe for the Genus, F) LEfSe for the Species. Alpha value for the factorial Kruskal-Wallis test used to do the difference statistic in abundance of phyla among groups. Threshold on the logarithmic LDA score (LDA score > 2) performed to estimate the abundance of discriminative taxa.
